# Defatted *Tenebrio molitor* Larva Fermentation Extract Modifies Steatosis, Inflammation and Intestinal Microflora in Chronic Alcohol-Fed Rats

**DOI:** 10.3390/nu12051426

**Published:** 2020-05-14

**Authors:** Ra-Yeong Choi, Ju Ri Ham, Hyo-Seon Ryu, Sang Suk Lee, Michelle A. Miguel, Man-Jeong Paik, Moongi Ji, Kyung-Wuk Park, Kyung-Yun Kang, Hae-In Lee, Mi-Kyung Lee

**Affiliations:** 1 Department of Food and Nutrition, Sunchon National University, Suncheon 57922, Korea; fkdud1304@naver.com (R.-Y.C.); punsu05@nate.com (J.R.H.); rhs0242@naver.com (H.-S.R.); 2 Department of Animal Science and Technology, Sunchon National University, Suncheon 57922, Korea; remen@scnu.ac.kr (S.S.L.); miguelmichelle18@yahoo.com (M.A.M.); 3 College of Pharmacy, Sunchon National University, Suncheon 57922, Korea; paik815@scnu.ac.kr (M.-J.P.); wlansrl@naver.com (M.J.); 4 Suncheon Research Center for Natural Medicines, Suncheon 57922, Korea; uk988446@nate.com (K.-W.P.); nms-kang@nate.com (K.-Y.K.); 5 Mokpo Marin Food-Industry Research Center, Mokpo 58621, Korea; hich2731@nate.com

**Keywords:** edible insect, gut microflora, inflammation, steatosis, *Tenebrio molitor*

## Abstract

This study examined the effects of defatted mealworm fermentation extract (MWF) on alcoholic liver injury in rats. The rats were fed either a Lieber-DeCarli control (Con) or alcohol liquid diet (EtOH). The alcohol-fed rats were administered MWF (50, 100, or 200 mg/kg/day) and silymarin (200 mg/kg/day) orally for eight weeks. MWF prevented alcohol-induced hepatocellular damage by decreasing their serum aspartate transaminase, alanine transaminase, and gamma-glutamyl transpeptidase levels significantly compared to the EtOH group. MWF effectively reduced the relative hepatic weight, lipid contents, and fat deposition, along with the down-regulation of transcriptional factors and genes involved in lipogenesis compared to the EtOH group. It also enhanced the antioxidant defense system by elevating the glutathione level and glutathione reductase activity. MWF attenuated the alcohol-induced inflammatory response by down-regulating hepatic inflammation-associated proteins expression, such as phosphorylated-inhibitor of nuclear factor-kappa B-alpha and tumor necrosis factor-alpha, in chronic alcohol-fed rats. Furthermore, sequencing analysis in the colonic microbiota showed that MWF tended to increase *Lactobacillus johnsonii* reduced by chronic alcohol consumption. These findings suggest that MWF can attenuate alcoholic liver injury by regulating the lipogenic and inflammatory pathway and antioxidant defense system, as well as by partially altering the microbial composition.

## 1. Introduction

Alcohol metabolism has numerous detrimental consequences, including the formation of acetaldehyde adducts and reactive oxygen species (ROS). In addition, it changes the ratio of the reduction and oxidation state of hepatocytes that contribute to the tissue damage and diseases observed in alcoholic patients [1]. The initiation and progression of alcoholic liver disease (ALD) occur through various etiologies, including oxidative stress, inflammation, dysbiosis, and metabolic dysregulation [2,3]. Hepatic steatosis is an initial manifestation of ALD in response to excess alcohol exposure and is characterized as excessive fat accumulation in liver cells [4]. Pharmacological treatments to improve ALD are still limited because of the side effects of synthetic medicines with hepatic protective activity. Therefore, stable and safe therapeutic strategies are needed to improve the progression of ALD for patients who do not stop drinking [5].

Previous studies have focused on the effects of non-toxic compounds extracted from natural food and herbal plants for ALD treatment [6,7]. Recently, the application of multiple amino acids, such as branched-chain amino acids (BCAAs), has been reported to have beneficial effects in various liver diseases [8,9]. The BCAAs-enriched mixture has been assessed as a means of protecting an alcoholic fatty liver and mitochondrial dysfunction [10]. Corsetti et al. [11] showed the essential amino acid-enriched mixture to ameliorate alcohol-induced liver damage.

Edible insects are lately quite considered as future food resources with high nutritional value and consumed in many countries including Asia, Oceania, Africa, and Latin America [12]. Numerous studies have investigated functional and pharmacological potential with increasing attention to edible insects [13]. *Tenebrio molitor* larva, as known as mealworm, appears to be a sustainable alternative protein source [14]. The mealworm is also rich in essential amino acids, polyunsaturated fatty acids, and a variety of vitamins and minerals, such as calcium, zinc, iron, magnesium, riboflavin, pantothenic acid, and biotin [15,16,17]. Previous studies reported that the *Tenebrio molitor* larvae possess pharmacological properties, such as anti-Alzheimer’s disease [18], anti-obesity [19], anticancer [20], anti-osteoporosis [21], anti-oxidation [22], and anti-inflammation activities [22]. Our previous study also showed that the defatted mealworm fermentation extract (MWF) has anti-oxidative effects against carbon tetrachloride-induced liver damage in mice [23]. On the other hand, little is known regarding the hepatic protective effects of MWF against alcohol-induced liver injury. Therefore, this study investigated the protective effects of MWF on chronic alcohol-induced liver injury and its underlying mechanism and explored the changes in intestinal microflora.

## 2. Materials and Methods

### 2.1. Preparation of Defatted Mealworm Fermentation Extract (MWF)

The *Tenebrio molitor* larval oil was extracted three times with edible hexane for 24 h at 24 °C and filtered through a paper filter (Whatman filter paper No. 2, Whatman plc, Maidstone, Kent, UK). Defatted *Tenebrio molitor* larvae were then concentrated using a rotary vacuum concentrator and freeze-dried. Subsequently, the concentrate was ground and used as an additive for fermentation. *Saccharomyces cerevisiae* strain (KCTC 17299) was purchased from the Korean Cell Line Bank (Seoul, Korea). The strain was inoculated at 1.5 mL and fermented at 32 °C with a stirring speed of 100 rpm for 72 h. The peptone in the composition of yeast-peptone dextrose medium (150 L) was replaced with defatted *Tenebrio molitor* larva powder. One liter of 70% fermented alcohol was added to 1 L of yeast/defatted *Tenebrio molitor* larva fermentation broth, and the mixture was extracted at 80 °C. The supernatant and precipitate were then separated. The extract was extracted by twice for 2 h, filtered through a paper filter (Whatman plc), and concentrated using a rotary vacuum evaporator. The extracted samples were evaporated and filtered using a 0.45 μm sterilized membrane filter. The fermented product was made of powder by freeze-drying and stored at −80 °C.

### 2.2. Animals and Experimental Design

Four-week-old male Sprague-Dawley rats were purchased from Orient Bio Inc. (Seoul, Korea). They were kept individually in stainless-steel cages in a temperature (20 ± 2 °C), humidity (50 ± 5%), and 12 h light/dark cycle-controlled room with access to food and water ad libitum. The Sunchon National University Institutional Animal Care and Use Committee approved the present study (SCNU_IACUC-2018-12).

After two weeks of acclimatization, the rats were divided randomly into the following six groups (*n* = 10 per group): (1) Con, control liquid diet, (2) EtOH, alcohol liquid diet, (3) MWF50, alcohol liquid diet + low-dosage MWF (50 mg/kg/day), (4) MWF100, alcohol liquid diet + medium-dosage MWF (100 mg/kg/day), (5) MWF200, alcohol liquid diet + high-dosage MWF (200 mg/kg/day), (6) Sily200, alcohol liquid diet + silymarin (200 mg/kg/day; Sigma-Aldrich, Co., St. Louis, MO, USA). The silymarin is known as the milk thistle and used as a dietary supplement for liver diseases in Asia, Southern Europe, and America [24]. The alcohol-fed rats were given a Lieber-DeCarli alcohol liquid diet for eight weeks by gradually increasing the alcohol content as described previously [25]. The Con group was pair-fed an isocaloric liquid diet containing dextrin-maltose instead of alcohol (Supplementary Table S1). At the beginning of the alcohol feeding, MWF and silymarin were well-dissolved in distilled water, respectively, and administered to the animals using oral zonde needle once a day in eight weeks. The rats in Con and EtOH groups were orally administered distilled water alone. The food intake and body weight were monitored daily and weekly, respectively.

At the end of the experiments, the animals were anesthetized with CO_2_ gas after fasting for 12 h. Blood was then harvested from the inferior vena cava. Serum was obtained by centrifuging the blood at 3000 rpm for 15 min at 4 °C. The livers and colons were removed and rinsed with PBS. Afterwards, the livers were weighed. The serum, liver, and colon samples were stored at −80 °C until analysis.

### 2.3. Dosage Information

Previous studies reported that the administration of freeze-dried powdered *Tenebrio molitor* larvae up to 3 g/kg/day to rats for 28 days or 90 days did not result in any adverse effects or toxicity [26,27]. Our previous research indicated that the consumption of 500 mg/kg/day MWF had no observable adverse effects on the mice for three days [23]. In this study, the MWF was administered orally to rats for eight weeks at a concentration of 50, 100, and 200 mg/kg/day, respectively. For the average 60 kg human, the doses translate into 480, 960, and 1920 mg/day, respectively, using an allometric scaling factor of 0.16.

### 2.4. Biochemical Analysis in Serum and Liver

The serum biochemistry examination was measured using Fuji Dri-Chem 3500i (Fujifilm, Tokyo, Japan) to measure the aspartate aminotransferase (AST), alanine aminotransferase (ALT), albumin, and total bilirubin levels. The serum gamma-glutamyl transpeptidase (γ-GTP) was measured using a commercial kit (YD Diagnostics Corp., Yongin, Korea). The serum tumor necrosis factor-alpha (TNF-α; Elabscience Biotechnology Co., Ltd., Wuhan, China) and interleukin-6 (IL-6; Aviva Systems Biology, San Diego, CA, USA) were determined using an ELISA kit. The serum triglyceride (TG), total cholesterol (TC), and HDL-cholesterol (Asan Pharmaceutical Co., Ltd., Seoul, Korea), and free fatty acid (FFA; Shinyang Diagnostics, Seoul, Korea) contents were determined according to the manufacturer’s instructions. The hepatic lipid contents (TG, FFA, and cholesterol) were measured using the same method as the serum after extraction, according to the Folch et al. [28].

### 2.5. Hepatic Histological Analysis

A piece of liver was fixed in 10% neutral buffered formalin, and embedded in paraffin. The liver sections (3–5 μm) were then stained with hematoxylin and eosin (H&E) and Masson’s trichrome. Frozen sections were stained with Oil Red O and viewed using an optical microscope.

### 2.6. RNA Isolation and Quantitative Real-Time PCR Analysis

The liver tissue was homogenized in TRIzol reagent (Invitrogen, Carlsbad, CA, USA), after which the total RNA was isolated according to the manufacturer’s instructions. The RNA quality was checked using a Nanodrop 2000 spectrophotometer (Thermo Fisher Scientific, Waltham, MA, USA). The total RNA (1 μg) was then reverse-transcribed into complementary DNA (cDNA) using a ReverTra Ace qPCR RT master mix (Toyobo, Osaka, Japan). Real-time PCR was performed using an SYBR green PCR kit (Qiagen, Hilden, Germany) and the CFX96 Touch^TM^ real-time PCR detection system (Bio-Rad Laboratories, Inc., Hercules, CA, USA). Table 1 lists the primers sequences. The data were analyzed using the 2^−△△CT^ method [29], and normalized to glyceraldehyde-3-phosphate dehydrogenase (GAPDH) in the same cDNA samples.

### 2.7. Western Blot Analysis

The liver tissue was homogenized in lysis buffer and centrifuged at 13,000 rpm and 4 °C for 30 min; the supernatants were used in further analysis. The total protein concentrations were determined using the Bradford method [30]. The protein samples (50 μg) were separated on a 10% SDS-PAGE and transferred to nitrocellulose membranes (Whatman plc). The membranes were probed with the primary antibodies against TNF-α, IL-6 (Santa Cruz Biotechnology, Inc., Dallas, TX, USA), phosphorylated-inhibitor of nuclear factor-kappa B-alpha (p-IκB-α; Cell Signaling Technology, Inc., Denvers, MA, USA), and β-actin (Sigma-Aldrich, St. Louis, MO, USA) overnight at 4 °C, and incubated with secondary antibodies (Cell Signaling Technology, Inc.) for 2 h. The protein bands were then visualized using an ECL reagent (Santa Cruz Biotechnology, Inc.) followed by brief exposure using an automated detection system (LAS 4000, Fujifilm, Tokyo, Japan). The amount of protein was quantified by densitometric analysis using the Multi Gauge program (Version 3.0, Fujifilm).

### 2.8. Hepatic Enzymatic Activities and Glutathione Level

Superoxide dismutase (SOD), catalase, and glutathione peroxidase (GSH-Px) activities were measured, as described previously [31]. The glutathione reductase (GR) activity was measured spectrophotometrically using a reaction mixture containing 770 μL of distilled water, 100 μL of 1 M potassium phosphate buffer (pH 7.4), 100 μL of 10 μM GSSG, 10 μL of 0.1 M EDTA, 10 μL of cytosolic fraction, and 10 μL of 10 mM NADPH. After reacting for two minutes at 25 °C, the change in absorbance was measured at 340 nm. The total GSH and reduced GSH contents were measured using commercial assay kits (DoGenBio Co., Ltd., Seoul, Korea).

### 2.9. Microbial Community Analysis in Colon

Three rats were randomly chosen from each group (Con, EtOH, MWF200, and Sily200). The colonic contents were collected into sterile tubes and stored at −80 °C. The DNA was extracted using a PowerSoil DNA isolation kit (MoBio Laboratories, Inc., Carlsbad, CA, USA) according to the manufacturer’s instructions. A sequenced sample was prepared according to the Illumina 16S Metagenomic Sequencing Library protocols. Subsequently, the 16S rRNA genes were amplified utilizing 16S V3–V4 primers. The resulting PCR products were normalized and pooled using the PicoGreen. The size of the libraries was verified using the TapeStation DNA screentape D1000 (Agilent Technologies, Inc., Santa Clara, CA, USA). Subsequently, the libraries were sequenced using the MiSeq™ platform (Illumina, San Diego, CA, USA), and the pair-end reads were assembled using FLASH (1.2.11) and analyzed by QIIME (v1.9) to perform the microbial community comparison. The bacterial community richness, evenness, and diversity were measured using the Chao1 index, Shannon index, and inverse Simpson index, respectively [32].

### 2.10. Statistical Analysis

Data are presented as the means ± standard error (SE). Statistical analysis was performed using the Statistical Package for the Social Sciences (SPSS version 25, SPSS Inc., Chicago, IL, USA). Data were analyzed by one-way ANOVA, followed by a Duncan’s multiple-range test. A *p* value < 0.05 was considered significant.

## 3. Results

### 3.1. MWF Ameliorated Alcoholic Hepatosteatosis

**Figure 1:**
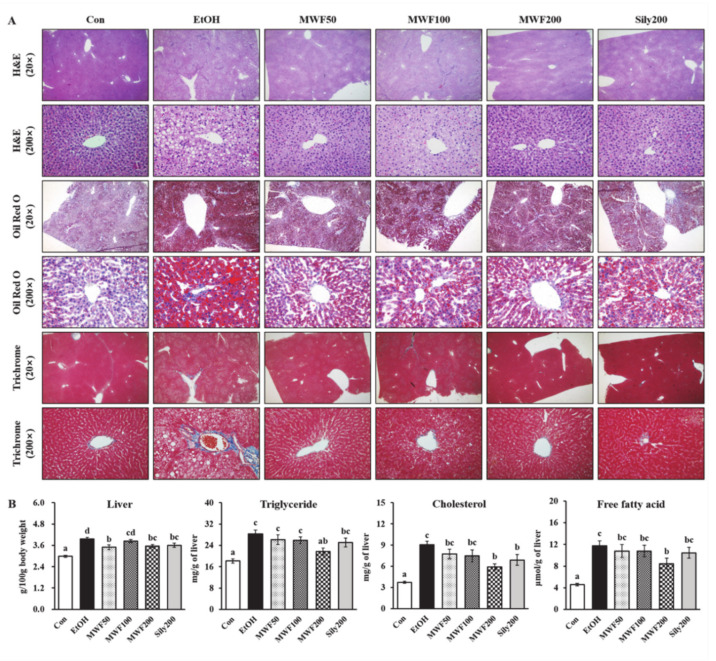
Effects of MWF on hepatic histopathological alterations (**A**), relative liver weight (**B**), and hepatic lipid contents (**B**) in chronic alcohol-fed rats. Mean ± SE. Statistical significance was determined by one-way ANOVA, followed by Duncan’s multiple range test. Values not sharing a common letter (a, b, c, d) above the bars are significantly different among the groups at *p* < 0.05.

H&E staining data showed that MWF (50, 100, and 200 mg/kg) and silymarin (200 mg/kg) reduced hepatic fat globules and focal necrosis compared with the EtOH group (Figure 1A). Oil Red O staining exhibited more hepatic lipid droplets in the EtOH group compared with the Con group. On the other hand, all doses of MWF and silymarin attenuated alcohol-induced hepatic lipid accumulation (Figure 1A) and also attenuated alcohol-induced mild fibrosis, as shown by trichrome staining (Figure 1A). The relative liver weight of the EtOH group was increased significantly as compared to the Con group, while MWF (50 and 200 mg/kg) and silymarin (200 mg/kg) sharply decreased it compared to the EtOH group (Figure 1B). The MWF200 group significantly decreased hepatic TG, cholesterol, and FFA levels by 23.2%, 34.5%, and 28.2%, respectively, compared to the EtOH group (Figure 1B). Not all doses of MWF and silymarin changed the serum albumin, total bilirubin, lipid contents, body weight, total body weight gain, and daily food intake (Supplementary Table S2). The serum AST, ALT, and γ-GTP activities in the EtOH group were increased markedly to approximately 4.0-, 6.1-, and 2.4-fold, respectively, compared with the Con group. However, these levels were significantly lower in both the MWF50 and MWF200 groups than in the EtOH group (Table 2).

### 3.2. MWF Down-Regulated Lipogenesis-Related Gene Expression

**Figure 2:**
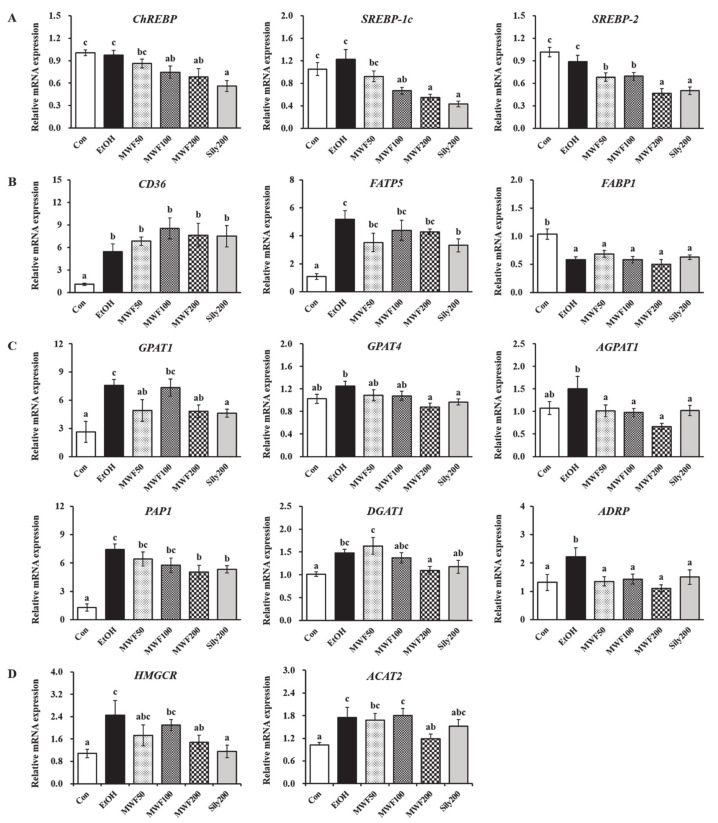
Effects of MWF on the hepatic lipogenesis-related gene levels in chronic alcohol-fed rats. (**A**) Lipid synthesis transcription factors; (**B**) fatty acid uptake and transport-related genes; (**C**) triglyceride synthesis-related genes; (**D**) cholesterol synthesis and esterification-related genes. Mean ± SE. Statistical significance was determined by one-way ANOVA, followed by Duncan’s multiple range test. Values not sharing a common letter (a, b, c) above the bars are significantly different among the groups at *p* < 0.05.

Considering carbohydrate-responsive element-binding protein (ChREBP), sterol regulatory element-binding protein (SREBP)-1c, and SREBP-2, the mRNA expressions of lipid synthesis transcription factors were decreased significantly in the MWF100, MWF200, and Sily200 groups compared to the EtOH group (Figure 2A). Chronic alcohol exposure significantly increased the cluster of differentiation 36 (CD36) and fatty acid transport protein 5 (FATP5) expression, but remarkably decreased fatty acid-binding protein 1 (FABP1) expression (Figure 2B). Silymarin (200 mg/kg) significantly reduced *FATP5* expression, whereas all doses of MWF slightly decreased *FATP5* expression compared to the EtOH group. Chronic alcohol consumption increased glycerol-3-phosphate acyltransferase (GPAT) 1, GPAT4, 1-acylglycerol-3-phosphate O-acyltransferase 1 (AGPAT1), phosphatidate phosphatase 1 (PAP1), and diacylglycerol O-acyltransferase 1 (DGAT1) gene expression; however, these changes were inhibited markedly in the MWF200 group by 36.6%, 29.9%, 55.7%, 32.1%, and 25.8%, respectively, compared to the EtOH group (Figure 2C). MWF (50, 100, and 200 mg/kg) and silymarin (200 mg/kg) exhibited a significant reduction of adipose differentiation-related protein (ADRP) expression on the surface of lipid droplets compared to the EtOH group (Figure 2C). As shown in Figure 2D, the EtOH group showed a significant up-regulation of 3-hydroxy-3-methylglutaryl-CoA reductase (HMGCR), cholesterol synthesis gene, and acetyl-CoA acetyltransferase 2 (ACAT2), cholesterol esterification gene. In contrast, their expression was reduced significantly in MWF200 group. The Sily200 group significantly down-regulated *GPAT1*, *GPAT4*, *AGPAT1*, *PAP1*, and *HMGCR* gene expression.

### 3.3. MWF Inhibited Alcohol-Induced Inflammatory Response

**Figure 3:**
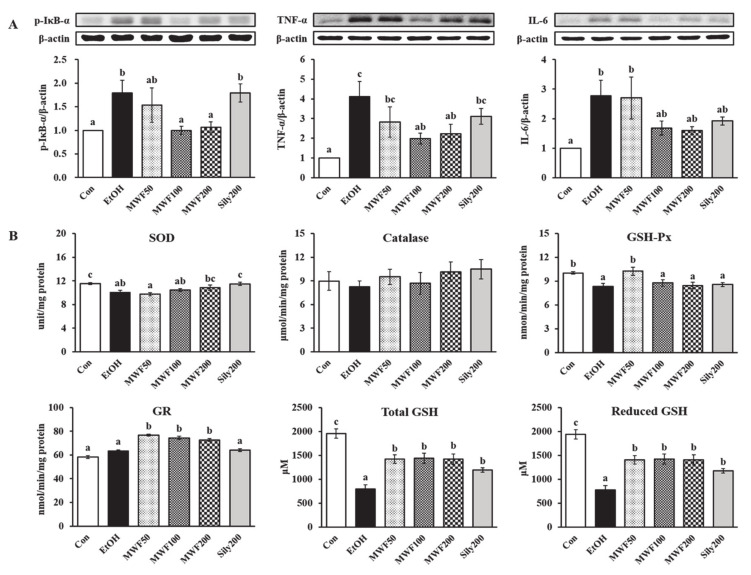
Effects of MWF on hepatic inflammatory protein expression (**A**), antioxidant enzymes activities (**B**), and glutathione concentrations (**B**) in chronic alcohol-fed rats. Mean ± SE. Statistical significance was determined by one-way ANOVA, followed by Duncan’s multiple range test. Values not sharing a common letter (a, b, c) above the bars are significantly different among the groups at *p* < 0.05.

The p-IκB-α protein expression of the EtOH group in the liver was higher than in the Con group, but it was decreased significantly in both MWF100 and MWF200 groups compared to the EtOH group (Figure 3A). The sily200 group did not affect p-IκB-α protein expression in the liver. The hepatic protein expression of TNF-α and IL-6 was increased remarkably by chronic alcohol consumption, but TNF-α was suppressed significantly in the MWF100 and MWF200 groups and IL-6 was reduced slightly compared to the EtOH group (Figure 3A). Serum TNF-α level was increased remarkably in the EtOH group compared to the Con group (Table 2). All doses of MWF decreased significantly in the serum TNF-α level compared to the EtOH group, and they slightly lowered serum IL-6 level without statistical significance.

### 3.4. MWF Reversed Alcohol-Induced Antioxidant Defense System Dysfunction

The SOD and GSH-Px activities in the EtOH group were lower than those in the Con group (Figure 3B). Although statistical significance was not observed, the MWF200 group tended to increase the SOD activity compared to the EtOH group. The GSH-Px activity was increased significantly in the MWF50 group compared to the EtOH group. The catalase activity did not show significant differences among the groups (Figure 3B). The GR activity was increased remarkably in the MWF50, MWF100, and MWF200 groups compared to the EtOH group (Figure 3B). Reduced and total GSH levels were significantly lower in the EtOH group than the Con group, but they were significantly increased by all doses of MWF and silymarin (Figure 3B).

### 3.5. MWF Altered the Bacterial Community Composition

**Figure 4:**
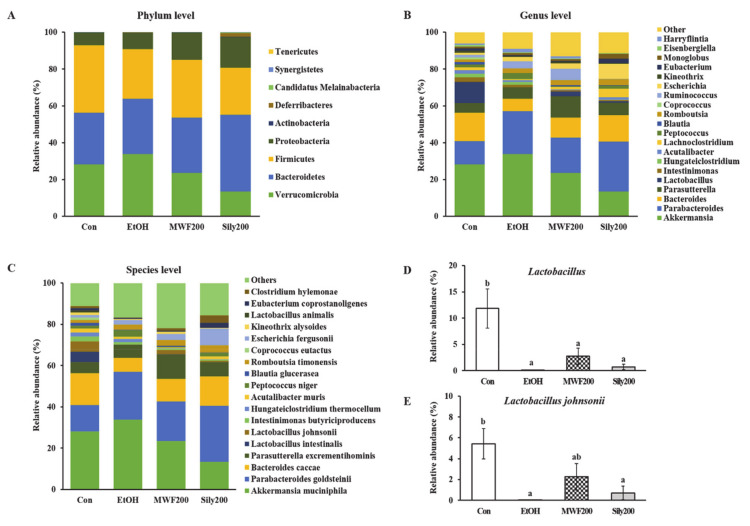
Effects of MWF on the bacterial community structures of the colon in chronic alcohol-fed rats. (**A**) Bacterial taxonomic composition at the phylum level; (**B**) genus level; (**C**) species level; (**D**) *Lactobacillus* genus; (**E**) *Lactobacillus johnsonii* species. Mean ± SE. Statistical significance was determined by one-way ANOVA, followed by Duncan’s multiple range test. Values not sharing a common letter (a, b) above the bars are significantly different among the groups at *p* < 0.05.

The alpha diversity indices did not change significantly among the groups (Table S3). The taxonomic classification yielded nine bacterial phyla, but the predominant phyla in all the groups were *Verrucomicrobia*, *Bacteroidetes*, and *Firmicutes* (Figure 4A). The phylum *Verrucomicrobia* was most dominant in the EtOH group (33.84%), whereas it was lowest in the Sily200 group (13.44%). The phylum *Bacteroidetes* was most dominant in the Sily200 group with a relative abundance of 41.64%, but the other groups had a similar ratio. The Con and MWF200 groups had the highest abundance of phylum *Firmicutes*, representing 36.69% and 31.39%, respectively. At the genus level, the MWF200 and Sily200 group increased the proportion of *Bacteroides*, *Parasutterella*, and *Lactobacillus* compared to the EtOH group, but that of *Akkermansia* oppositely decreased (Figure 4B). In particular, the relative abundance of genus *Lactobacillus* was significantly lower in the EtOH group than in the Con group, on the other hand, the MWF200 group showed a 40.5-fold increase compared to the EtOH group. Unfortunately, the difference was not significant (Figure 4D). *Lactobacillus* species was almost undetectable in the EtOH group, but the relative abundance of *Lactobacillus johnsonii* species was 141.4-fold higher in the MWF200 group (Figure 4C,E).

## 4. Discussion

Recently, edible insects have been evaluated for their efficacy in relieving alcoholic liver damage [13,33]. Previous studies reported that essential amino acids, glutamic acid, or aspartic acid recovered the alcohol-induced liver toxicity [11,34]. A fermented defatted *Tenebrio molitor* larvae broth increased the essential amino acid concentration time-dependently until 72 h (data not shown). The enriched amino acids in MWF could protect ALD, and suggests for the first time, that MWF effectively attenuates hepatosteatosis in chronic alcohol-fed rats.

The present study examined the effects of MWF on hepatic lipid synthesis-related gene expression to understand the underlying mechanisms. Yu et al. [35] established that *ChREBP* and *SREBP-1c* mRNA expression increased in alcohol-induced early hepatic steatosis, and then increased FA uptake and TG synthesis. In this study, we did not find the significant changes of *ChREBP* and *SREBP-1c* mRNA levels caused by alcohol. However, MWF (100 and 200 mg/kg) and silymarin (200 mg/kg) down-regulated *ChREBP*, *SREBP-1c*, and *SREBP-2* gene expression significantly. SREBP-1c and ChREBP are the major transcriptional factors that promote lipogenesis [36], and SREBP-2 mainly induces cholesterol synthesis and uptake in the liver [37].

GPAT is responsible for the first and rate-limiting step in TG synthesis. Although the direct effects of ChREBP on GPAT expression have not been clearly established, SREBP-1 directly controls GPAT expression [38]. The present study also found that the MWF or silymarin (200 mg/kg) decreased alcohol-induced TG (*GPAT1*, *GPAT4*, *AGPAT1*, *PAP1*, and *DGAT1*) and cholesterol (*HMGCR* and *ACAT2*) synthesis significantly, which resulted in reduced hepatic TG and cholesterol contents. In particular, ADRP is a reliable and sensitive marker for lipid droplets in alcoholic fatty liver [39]. The *ADRP* down-regulation by MWF and silymarin might be associated with the inhibition of hepatic fat deposition. The alcohol intake increases hepatic TG by increasing the uptake of exogenous FFA [40,41,42]. Long chain fatty acid uptake is dependent on FATPs, FABPs, and CD36/FAT [43,44]. FATP5 knockout mice decreased the hepatic TG and FFA levels [44,45]. In the present study, *CD36* and *FATP5* expression were increased in alcohol-fed rats, whereas *FABP1* expression was decreased. Silymarin down-regulated *FATP5* expression, whereas MWF tended to decrease *FATP5* expression compared to the EtOH group. As the results, the anti-steatotic effects of MWF might be mediated through suppression of de novo TG and cholesterol synthesis rather than regulating exogenous FFA uptake in the liver.

Animal models of ALD and patients with alcoholic hepatitis showed an increase in the TNF-α, IL-1, and IL-6 levels. Distinctly, alcoholic liver injury is considered to be mediated by TNF-α [46,47]. In the current study, MWF decreased the serum TNF-α, its hepatic protein expression, and its up-stream p-IκB-α protein expression induced by alcohol. Lin et al. [48] reported that pepsin-digested chicken liver hydrolysates (80, 320, and 1280 mg/kg) or silymarin (150 mg/kg) ameliorated the hepatic TNF-α and IL-6 levels in alcoholic diet fed mice for eight weeks. Nuclear factor-kappa B (NF-κB) binds to IκB and presents in an inactive state [49]. In response to stimulation, such as oxidative stress and inflammatory cytokines, IκB-α is phosphorylated by IκB-kinase (IKK) phosphorylation, and subsequently, IκB-α/NF-κB complex degradation leads to the activation and release of NF-κB [49]. Herein, MWF attenuated alcohol-induced inflammation by inhibiting the NF-κB pathway. The inflammatory response is also characterized by infiltration of the liver with immunocytes, and these data reflect the enlarged liver (hepatomegaly) and serum liver damage (AST, ALT, and γ-GTP) [48]. The present study found that MWF (200 mg/kg) effectively improved the relative liver weight and serum liver damage markers. Sun et al. [50] formerly informed that hydrolysates from corn gluten meal that were rich in BCAAs lowered the AST and ALT activities in an acute alcoholism model. Consistent with these results, previous studies reported that animal-derived protein hydrolysates reduced the serum AST and ALT levels as well as the macro-lipid droplets and TG levels in the liver of chronic alcohol-fed mice [48,51].

Hepatic steatosis commonly results in excessive ROS generation, which plays a critical role in the pathogenesis of ALD [52]. The antioxidant defense system for balancing the intracellular oxidative stress consists of enzymatic antioxidants (SOD, catalase, GSH-Px, and GR) and non-enzymatic antioxidants (GSH) [53]. In our study, the total and reduced GSH levels in the EtOH group were decreased by 59.8% compared to the Con group, but MWF increased these levels to above 80.1% compared to the EtOH group. Hepatic GSH depletion above 20% has been observed to impair the cellular antioxidant defenses against ROS and causes hepatic injury [54,55]. A previous study showed that the supplementation of cysteine, glycine, and glutamic acid increased the GSH level [56]. In the current study, the increase in GSH content is believed to be influenced by the amino acids contained in MWF. The GR activity was remarkably increased at whole MWF doses compared to the EtOH group, which indicated that it is involved in the antioxidant mechanisms by increasing the reduction to GSH from GSSG. Je et al. [57] presented that alcohol intake for four weeks decreased significantly the hepatic GSH content as well as the hepatic SOD and GSH-Px activities, whereas peptic hydrolysates from salmon pectoral fin protein byproducts recovered these parameters [57]. Similarly, MWF200 tended to increase the SOD activity, and MWF50 increased the GSH-Px activity significantly compared to the EtOH group in the liver, which showed that the anti-oxidative defense capacity might originate from the differences in the animal age, applied ethanol dose, and experimental duration [58]. These results suggest that the MWF positively reacts with the hepatic antioxidant defense system to reverse alcohol toxicity.

Alcohol induces quantitative and qualitative alterations in the gut microbiota and increases the gut permeability [3]. Therefore, this study examined whether MWF200 affects the alcohol-induced changes in the bacterial communities in the colon. This study demonstrated that alcohol decreased the proportion of *Firmicutes* phylum and increased the relative abundance of *Bacteroidetes* and *Verrucomibrobia* phyla. These outcomes are consistent with the previous finding from mice that received alcohol for three weeks [59]. In contrast, Bull-Otterson et al. [60] reported that alcohol decreased the *Bacteroidetes* phylum and increased the *Firmicutes*, *Proteobacteria*, and *Actinobacteria* phyla in stool of mice fed a Lieber-DeCarli liquid diet for eight weeks. They suggest that the intestinal bacterial composition can be influenced by the duration of alcohol consumption, animal age, species, and sample type (intestine or stool). Interestingly, our current study showed that *Lactobacillus* species was depleted in the EtOH group, but the abundance of *Lactobacillus* was slightly increased in the MWF200. MWF specially showed a tendency to increase the abundance of *Lactobacillus johnsonii* compared to the EtOH group. The alcohol intake in rats was associated with the reduction of lactic acid bacteria: the genera *Lactobacillus*, *Pediococcus*, *Leuconostoc*, and *Lactococcus* [59]. Leclercq et al. [61] reported a decrease in beneficial bacteria, such as *Lactobacillus* and *Bifidobacterium*, in animals exposed to alcohol and in alcohol-dependent subjects. These bacteria’s restoration may be a potential therapeutic target for alcohol-related diseases. Thus, the restorative effects of MWF on the alcohol-induced gut microflora changes might be associated with the increased *Lactobacillus johnsonii* abundance.

## 5. Conclusions

**Figure 5:**
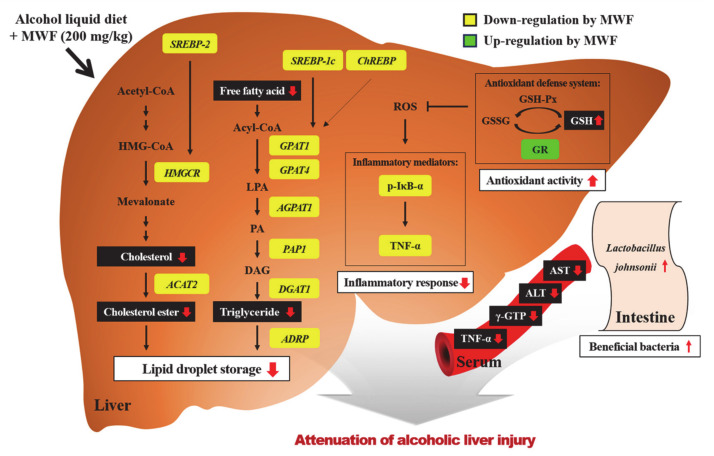
Proposed mechanisms for protective effects of MWF on chronic alcohol-induced liver injury. MWF decreased gene expression involved in de novo TG and cholesterol synthesis, which may contribute to anti-steatotic effects. Moreover, MWF reduced alcohol-induced inflammatory response by inhibiting the NF-κB pathway. MWF positively reacts with the hepatic antioxidant defense system as well as protectively alters alcohol-induced intestinal microflora changes.

The beneficial effects of MWF on alcohol-induced liver injury may be mediated partially by modulating hepatic lipid synthesis, inflammation, and GSH contents. These protective effects of MWF200 have the most potential among MWF50, MWF100, and Sily200. The indirect effects of MFW on liver also might be mediated by alterations of gut microbiota, which will be further elucidated in future works. Figure 5 illustrates the possible mechanisms for protective effects of MWF on alcohol-induced liver injury. Overall, these results suggest that MWF could potentially be developed as a valuable source of functional food ingredients for protecting against alcoholic liver injury.

## Figures and Tables

**Table 1:** Primer sequences for real-time PCR.

Gene	Full Name	Forward/Reverse(5′–3′)
*ACAT2*	Acetyl-CoA acetyltransferase 2	CGGTTTGATTCGAGCACCAC/ACTTTGATGGCCCCTGTTCC
*ADRP*	Adipose differentiation-related protein	CCTATCATCAGGCTCTCGGC/GTGCACATTCTTCCTGGCGA
*AGPAT1*	1-Acylglycerol-3-phosphate O-acyltransferase 1	AGGACATCCCCAAATCCTGTCG/GCCACAGCTCCATTCTGGTCA
*CD36*	Cluster of differentiation 36	TCCTCGGATGGCTAGCTGATT/TGCTTTCTATGTGGCCTGGTT
*ChREBP*	Carbohydrate-response element-binding protein	TATGCCGGGACAAGATTCGG/AGGTTTCCGGTGCTCATCTG
*DGAT1*	Diacylglycerol O-acyltransferase 1	CAGCAGTGGATGGTCCCTAC/ACCGCCAGCTTTAAGAGACG
*FABP1*	Fatty acid-binding protein 1	CTTCTCCGGCAAGTACCAAGT/CATGCACGATTTCTGACACCC
*FATP5*	Fatty acid transport protein 5	CTGAGGGCTGCTCAATCACA/GTGGCTTCCTTCAGCTTTGC
*GAPDH*	Glyceraldehyde-3-phosphate dehydrogenase	AGTGCCAGCCTCGTCTCATA/ATGAAGGGGTCGTTGATGGC
*GPAT1*	Glycerol-3-phosphate acyltransferase 1	ACCACATCAAGGATACAGCTC/CCTCATTCGTGTGTTTACATCGG
*GPAT4*	Glycerol-3-phosphate acyltransferase 4	TGTGGGACGGTGGATTGAAG/GCTCCGGTCCTCATGGTTAC
*HMGCR*	3-Hydroxy-3-methylglutaryl-CoA reductase	CCTCCATTGAGATCCGGAGG/GATGGGAGGCCACAAAGAGG
*PAP1*	Phosphatidate phosphatase 1	TCACTACCCAGTACCAGGGC/TGAGTCCAATCCTTTCCCAG
*SREBP-1c*	Sterol regulatory element-binding protein 1c	GACGAGCTACCCTTCGGTG/GGGGCATCAAATAGGCCAGG
*SREBP2*	Sterol regulatory element-binding protein 2	CGAACTGGGCGATGGATGAGA/TCTCCCACTTGATTGCTGACA

**Table 2:** Effects of MWF on the serum marker levels in chronic alcohol-fed rats.

	Con	EtOH	MWF50	MWF100	MWF200	Sily200
**TNF-α (pg/mL)**	54.74 ± 5.42 ^a^	74.66 ± 6.05 ^b^	53.87 ± 4.85 ^a^	42.52 ± 6.07 ^a^	45.55 ± 2.61 ^a^	46.06 ± 9.14 ^a^
**IL-6 (pg/mL)**	3.03 ± 0.30	3.03 ± 0.50	2.90 ± 0.39	2.19 ± 0.30	1.87 ± 0.12	2.36 ± 0.27
**γ-GTP (U/L)**	21.06 ± 4.31 ^a^	50.44 ± 6.84 ^c^	29.55 ± 3.92 ^ab^	35.61 ± 3.03 ^b^	38.48 ± 3.46 ^b^	37.41 ± 2.92 ^b^
**AST (U/L)**	65.10 ± 1.47 ^a^	259.83 ± 42.84 ^c^	162.12 ± 17.94 ^b^	189.45 ± 16.37 ^bc^	172.88 ± 32.75 ^b^	184.38 ± 32.70 ^bc^
**ALT (U/L)**	28.50 ± 2.26 ^a^	173.94 ± 40.95 ^c^	90.12 ± 15.46 ^ab^	118.50 ± 13.49 ^bc^	95.22 ± 18.29 ^ab^	131.33 ± 36.38 ^bc^

Mean ± SE. Statistical significance was determined by one-way ANOVA, followed by Duncan’s multiple range test. Values not sharing a common letter (a, b, c) in the same row are significantly different among the group at *p* < 0.05.

## Supplementary Materials

The following are available online at ’https://www.mdpi.com/2072-6643/12/5/1426/s1, Table S1: Composition of the control and alcohol liquid diets, Table S2: Effects of MWF on the body weight, food intake, serum marker levels, and lipid contents in chronic alcohol-fed rats, Table S3: Effects of MWF on the richness and diversity of the bacterial community deduced by 16S rDNA profiling in chronic alcohol-fed rats
