# Supplementary material for: Defatted Tenebrio molitor Larva Fermentation Extract Modifies Steatosis, Inflammation and Intestinal Microflora in Chronic Alcohol-Fed Rats
Source: Nutrients. 2020 May 14;12(5):1426. doi: 10.3390/nu12051426 (PMC7284378; doi:10.3390/nu12051426)
Supplement: Supplementary file 1 [file nutrients-12-01426-s001.pdf]

MWF altered the bacterial community composition

The alpha diversity indices showed no significant differences among the groups. The EtOH group contained the highest number of operational taxonomic units (OTUs), whereas the lowest was observed in the Sily200 group. In terms of the Shannon index, the microbial diversity in the MWF200 group was lower than that in the Con and Sily200 groups but comparable to the EtOH group. The inverse Simpson diversity was highest in the MWF200 group and lowest in the EtOH group. Good's coverage of all treatments ranged from 99.94% to 100%, suggesting that the sequencing used effectively characterizes the microbial composition. No differences in the Good's coverage index were observed, suggesting that sequencing coverage was similar among the groups.

References

1. Chao, A. Nonparametric Estimation of the Number of Classes in a Population. *Scand. J. Stat.* **1984,** *11,* 265-270.
2. Shannon, C.E. A Mathematical Theory of Communication. *Bell Syst. Tech. J.* **1948,** *27,* 379-423.

Table S1. Composition of the control and alcohol liquid diets [1]

(g/L)

Ingredient	Control liquid diet [2]	Alcohol liquid diet
Casein	41.4	41.4
L-Cystine	0.5	0.5
DL-Methionine	0.3	0.3
Corn oil	8.5	8.5
Olive oil	31.1	31.1
Dextrin maltose	115.2	25.6
Choline bitartrate	0.53	0.53
Cellulose	10.0	10.0
Xanthan gum	3.0	3.0
Vitamin Mixture [3]	2.55	2.55
Mineral Mixture [4]	9.0	9.0
Ethanol	-	50

[1] The liquid diet was mixed in 1 L of distilled water.
[2] The alcohol in the alcohol liquid diet was replaced with additional dextrin maltose in the Con group.
[3] AIN-76 vitamin mixture.
[4] AIN-76 mineral mixture.

Table S2. Effects of MWF on the body weight, food intake, serum marker levels, and lipid contents in chronic alcohol-fed rats

	Con	EtOH	MWF50	MWF100	MWF200	Sily200
**Body weight (g)**						
**Initial**	232.03±3.64	232.63±3.89	232.03±3.43	232.09±3.45	232.29±3.36	232.23±3.31
**Final**	485.99±5.34^{c}	423.48±12.49^{ab}	400.58±12.81^{a}	425.74±12.98^{ab}	407.38±10.82^{ab}	436.89±9.23^{b}
**Total body weight gain (g)**	253.95±5.26^{c}	190.85±10.36^{ab}	168.54±11.48^{a}	193.64±12.14^{ab}	175.08±9.11^{ab}	204.65±7.55^{b}
**Food intake (mL/day)**	85.18±0.12	84.58±1.35	80.78±2.22	84.43±2.57	78.64±1.81	84.65±2.17
**Serum marker levels**						
**Albumin (g/dL)**	4.04±0.09^{a}	4.47±0.11^{b}	4.40±0.08^{b}	4.49±0.07^{b}	4.41±0.10^{b}	4.45±0.14^{b}
**Total bilirubin (mg/dL)**	0.17±0.04	0.23±0.04	0.24±0.04	0.19±0.03	0.21±0.05	0.17±0.02
**Serum lipid contents**						
**Triglyceride (mg/dL)**	91.82±17.45^{b}	43.30±3.40^{a}	43.62±3.20^{a}	58.25±6.19^{a}	43.20±3.18^{a}	47.24±5.49^{a}
**Total cholesterol (mg/dL)**	82.70±5.75^{a}	106.70±8.91^{b}	106.05±7.42^{b}	109.95±6.25^{b}	120.10±7.20^{b}	128.90±9.64^{b}
**Free fatty acid (mmol/L)**	0.445±0.047	0.438±0.021	0.469±0.046	0.473±0.025	0.421±0.025	0.357±0.029
**HDL-cholesterol (mg/dL)**	55.29±3.53^{a}	68.01±5.91^{ab}	63.25±6.94^{ab}	74.22±5.76^{ab}	79.76±6.19^{b}	81.18±8.13^{b}

Mean ± SE. Values not sharing a common letter (a, b, c) in the same row are significantly different among the groups.

Table S3. Effects of MWF on the richness and diversity of the bacterial community deduced by 16S rDNA profiling in chronic alcohol-fed rats

Items	OTUs[1)]	Chao1[2)]	Shannon (H′)[3)]	Inverse Simpson[4)]	Good's coverage
**Con**	120.33±20.62	137.41±23.93	3.66±0.19	0.841±0.020	99.94±0.009
**EtOH**	120.66±5.23	146.98±4.56	3.43±0.29	0.817±0.020	99.94±0.011
**MWF200**	109.33±11.34	120.10±6.16	3.42±0.09	0.853±0.016	99.97±0.002
**Sily200**	99.66±13.54	112.16±19.52	3.51±0.24	0.846±0.028	100.0±0.037

[1] Operational taxonomic units.

[2] Chao1 richness estimator: an estimation of the total number of OTUs present in the community. A higher number indicates a higher richness [1].

[3] Shannon index (H′): a community diversity index that combines the number of OTUs present and their distribution [2].

[4] Inverse Simpson: the probability that two randomly selected individuals in the habitat will belong to the same species.
